# Pseudotumor Cerebri Secondary to Jugular Bulb Thrombosis: A Case Report and a Review of the Diagnostic Steps

**DOI:** 10.7759/cureus.27557

**Published:** 2022-08-01

**Authors:** Gyusik Park, Mohamad Fleifel, Hassan N Kesserwani

**Affiliations:** 1 Neurology, University of Alabama at Birmingham Marnix E. Heersink School of Medicine, Birmingham, USA; 2 Internal Medicine, American University of Beirut Medical Center, Beirut, LBN; 3 Neurology, Flowers Medical Group, Dothan, USA

**Keywords:** headache, cerebral venous thrombosis, jugular bulb thrombosis, idiopathic intracranial hypertension, pseudotumor cerebri

## Abstract

Pseudotumor cerebri (PTC) secondary to cerebral venous sinus thrombosis can be a difficult diagnosis to make for various reasons, including an atypical patient profile and potentially pleomorphic signs and symptoms. The symptoms can be insidious and can evolve acutely, subacutely, or chronically. To complicate the picture even further, neurodiagnostic testing can be particularly troublesome due to both false-positive and false-negative results. Frequently, multiple imaging modalities are variably deployed, and they include computed tomography (CT) with and without contrast, computed tomography venogram (CTV), magnetic resonance imaging (MRI), and magnetic resonance venography (MRV) of the brain. The thrombus can be quite subtle, requiring the seasoned eye of an experienced neuroradiologist. Nevertheless, when a diagnosis is made, the treatment can be highly efficacious and gratifying as it can prevent serious visual complications. We present a rare case of PTC due to a jugular bulb thrombosis and outline the challenging diagnostic steps.

## Introduction

Idiopathic intracranial hypertension (IIH) and PTC are terms frequently used interchangeably. Strictly speaking; however, the latter is usually associated with an identifiable secondary cause such as cerebral venous thrombosis or a trigger, including the use of minocycline or tetracycline. IIH, as the name implies, is idiopathic without an obvious etiology and is mostly a disease of young obese women [1].

Both conditions are associated with elevated cerebrospinal fluid (CSF) pressure. The modified Dandy criteria used for the diagnosis of benign intracranial hypertension require the following: 1) an elevated CSF pressure of greater than 250 mm water in an adult and 280 mm water in a child with non-inflammatory CSF findings, 2) signs and symptoms of elevated CSF pressure such as headaches, blurring of vision, transient visual obscurations, aggravation of headaches with supine posture, or diplopia, 3) the absence of focal neurological findings except for an abducens nerve palsy, 4) the absence of structural brain lesion or ventriculomegaly, 5) the presence of variable radiological findings, including an empty sella turcica, optic nerve distension with and without tortuosity of the orbital optic nerve, flattening of the lamina cribrosa at the optic nerve head, or a possible cerebral venous sinus stenosis in the absence of thrombosis, and 6) ophthalmological evidence of optic disc edema [2,3]. The term PTC is reserved for patients with an underlying etiology, one of the most frequent being cerebral venous sinus thrombosis.

In Walter Dandy’s reports of 22 patients with elevated intracranial pressure, none of the patients had ventriculomegaly, and all had a chronic clinical course that improved with decompressive craniotomy [2]. Based upon these clinical characteristics, he posited that the etiology of PTC was elevated intracranial pressure in the absence of visible ventricular obstruction and cerebral edema, as they usually follow an acute clinical course. More recently, the role of cerebral venous sinus thrombosis, microgravity-induced cephalad shift of CSF, aquaporin-4 receptors on astrocytes, and the choroid plexus sodium/potassium adenosine triphosphatase (ATPase) pump have been explored to further shed light on the pathogenesis of IIH and PTC [4]. Antibiotics minocycline/tetracycline and carbonic anhydrase inhibitors seem to modulate the activity of aquaporin-4 receptors and sodium/potassium ATPase [4].

## Case presentation

The patient is a 22-year-old woman who was referred from the ophthalmologist’s office to the emergency department with a two-week history of a severe global headache associated with transient visual obscurations described as her vision going dark for a few seconds when she stands up quickly. She also noted a blind spot in the right half of her visual field and a roaring sound in both ears. Noteworthy is the fact that she had gained 30 pounds in weight over the last six months. Her past medical history did not reveal any instances of miscarriage, deep venous thrombosis, or cerebral venous thrombosis. The patient denies any use of oral contraceptive pills. Her family history did not disclose any deep venous thrombosis or cerebral venous thrombosis. She was a healthy young woman with no recent exposure to antibiotics such as tetracycline or minocycline.

On examination, the patient was in mild distress. Her blood pressure was measured at 129 mmHg systolic and 79 mmHg diastolic with a pulse of 83 beats per minute. She was afebrile with a height of five feet and six inches, a weight of 218 pounds, and a body mass index of 38.6 kg/m^2^. Her gait was of normal stability and cadence. Cranial nerve examination revealed full ocular motion without an abducens nerve palsy. Pupillary reflexes were brisk to direct and consensual light stimulation with normal accommodative reflexes. The visual acuity was noted to be 20/50 in the right eye and 20/25 in the left eye. The fundoscopic exam revealed bilateral optic disc edema without any retinal nerve fiber layer defects. It also showed absent venous pulsations with the blurring of disc margins without optic disc pallor. Visual perimetry was not performed, and the optic disc edema was not graded. The rest of the cranial nerve examination was normal. The rest of the neurological evaluations involving motor strength, sensation, deep tendon reflex, and coordination were normal in all extremities without any focal deficits.

**Figure 1:**
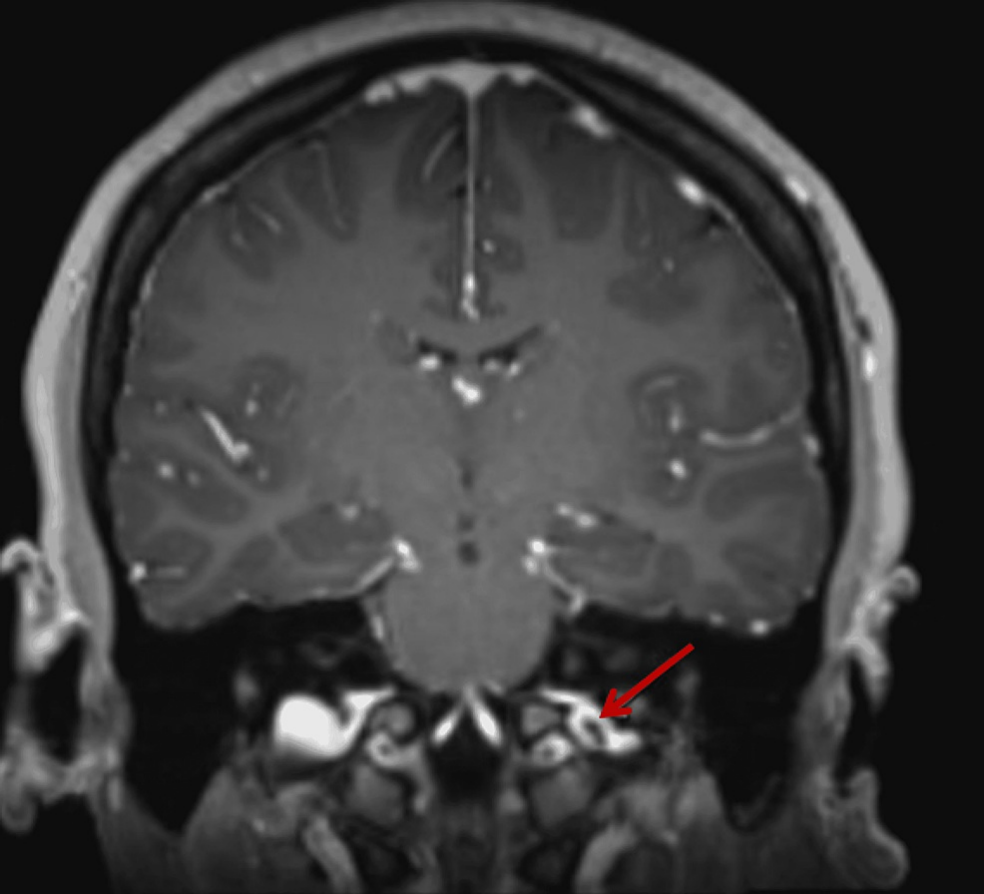
T-1 weighted coronal section MRI of the brain demonstrating a non-occlusive thrombus in the left IJV at the level of the jugular bulb at the skull base (red arrow)

**Figure 2:**
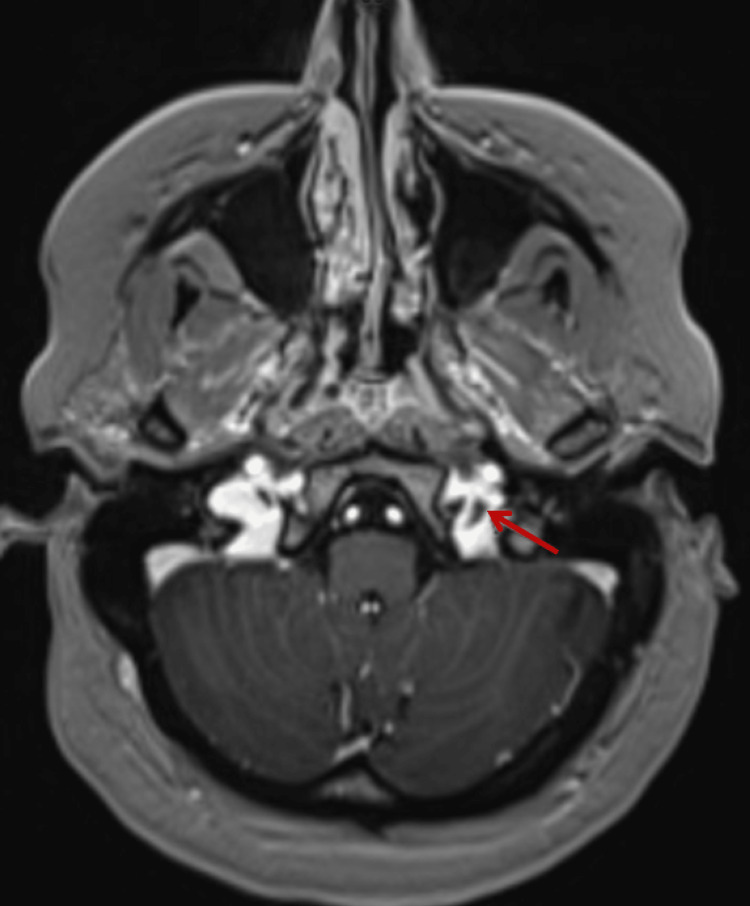
Axial MRI of the head and neck demonstrating a filling defect in the left jugular bulb (red arrow)

After an initial normal CT scan of the brain, a lumbar puncture was performed in the lateral decubitus position. Opening pressure was 36 cm water, and closing pressure was 21 cm water after drainage of 12 ml of spinal fluid. CSF analysis was non-inflammatory with 0 white blood cells, eight red blood cells, a glucose level of 59 mg/dL (normal: 40-70 mg/dL), and a protein level of 27 mg/dL (normal: 10-46 mg/dL). MRI of the brain showed a non-occlusive thrombus in the left internal jugular vein (IJV) at the level of the jugular bulb at the skull base, along with narrowing and stenosis of the bilateral distal transverse sinuses (Figure 1). MRI of the head and neck showed a subtle filling defect in the left jugular bulb region and narrowing of the bilateral distal transverse sinuses with the venous phase (Figure 2).

A comprehensive thrombophilia panel that includes serum lupus anticoagulant, serum anti-phospholipid antibodies, serum homocysteine, activated protein C (APC) resistance, prothrombin mutation, anti-thrombin III activity, protein C, and protein S antigen levels, platelet count, and serum beta-2 microglobulin levels was normal. A serum pregnancy test was negative. Based on the clinical symptomatology, bilateral optic disc edema, elevated CSF pressure with non-inflammatory CSF findings, and radiological findings, the patient was diagnosed with PTC secondary to left IJV thrombosis at the jugular bulb. She received acetazolamide 250 mg thrice daily. She also received weight-adjusted therapeutic enoxaparin and was later transitioned to oral apixaban 20 mg daily upon discharge. At the neurology clinic four days later, she reported near-complete resolution of the headaches and complete resolution of the tinnitus and visual obscurations. Visual acuity was 20/20 in both eyes.

## Discussion

IIH and PTC usually manifest with frequent frontal and retrobulbar headaches (93%-99%) with associated nausea, photophobia and phonophobia (71.5%), monocular or binocular transient vision loss (70%), persistent vision loss (30%), and unilateral or bilateral pulsatile tinnitus (60%) [5-7]. Ocular examination findings include optic disc edema, visual field loss, and abducens nerve palsy [6,7]. Our patient presented with the classical symptoms of headaches, transient visual obscurations, peripheral vision loss, and bilateral tinnitus. She also had bilateral optic disc edema and a significantly elevated CSF pressure, cardinal features of IIH/PTC.

PTC with IJV thrombosis is an entity that has been reported in the literature. Konrad et al. document the case of a 39-year-old man with a diagnosis of left IJV thrombosis who presented with acute diplopia and transient visual obscurations [8]. The case was considered noteworthy due to three uncommon aspects: 1) occurrence in a male, 2) unilateral IJV thrombosis, and 3) the lack of an underlying coagulation disorder. Another reported case was of a 45-year-old man with a history of laryngeal squamous cell carcinoma status post radiotherapy and cetuximab who presented with headaches and left eye vision loss [9]. The diagnosis of PTC with complete occlusion of the right IJV was made with the postulated underlying cause of cerebral venous drainage insufficiency from the nondominant left transverse sinus (opposite to a dominant right transverse sinus). Our case had similar clinical manifestations but with a radiological finding of jugular bulb thrombosis. Although the patient in our case also had bilateral transverse stenosis, which has been commonly described in patients with PTC, the stenosis developed likely secondary to increased intracranial pressure rather than being the primary cause of the PTC. This is supported by previous reports that describe the partial resolution of the stenosis with CSF removal [10,11].

Cerebro-cervical thrombosis has been described at multiple levels extending from the jugular foramen to the junction of the IJV and the subclavian vein. The jugular bulb, located in the jugular fossa, is the convergence point of the lateral venous sinuses. It drains to the IJV extracranially. IJV thrombosis is a life-threatening condition that could potentially lead to intraluminal thrombus propagation. IJV thromboembolism can lead to many complications, including PTC, pulmonary embolism, superior vena cava syndrome, septic emboli, and sagittal sinus thrombosis [12]. Etiologies can include hypercoagulable states, polycythemia vera, malignancy, regional infections, intravenous drug abuse, trauma, and iatrogenic causes such as head and neck surgery or central venous catheter placement [13].

For radiologic detection of IJV thrombosis, a head CT with or without contrast may show a hyperdensity of a cortical vein or a filling defect with the exclusive enhancement of the venous sinus lining [13]. A CTV is another fast modality test that is highly sensitive to subacute or chronic cases [13]. In the acute stages of thrombus formation defined as less than five days, MRV can serve as an important tool in early detection; it outlines the hypointensity on T2-weighted images, which reflects the deoxyhemoglobin state of red blood cells in a thrombotic lesion [13,14]. Invasive cerebral angiography is reserved for inconclusive non-invasive imaging, or if an endovascular intervention is planned, it would demonstrate a venous phase filling defect in the thrombosed cerebral vein [13].

The mainstay of therapy for IJV thrombosis is anticoagulation, including low-molecular-weight heparin (LMWH), unfractionated heparin (UFH), vitamin K antagonists, and direct oral anticoagulants (DOACs). The treatment duration is similar to that of pulmonary embolism and can range from 3-12 months to longer depending on individual risks; life-long treatment may be required if there is an underlying thrombophilia [15,16]. Thrombolysis is not recommended as first-line therapy due to the high risk of hemorrhage and insufficient evidence for its effectiveness [17]. Thrombolysis is only indicated if all the following criteria are met: 1) severe symptoms, 2) onset within 14 days, 3) long thrombus extent, 4) good individual performance status, 5) low bleeding risk, and 6) life expectancy greater than one year [18].

## Conclusions

Our case highlights the cascade of events that can happen when a patient with suspected PTC and cerebral venous thrombosis is encountered. It demonstrates the importance of being vigilant to the possibility of cerebral venous thrombosis, especially when the patient does not fit the typical profile of IIH/PTC and whenever visual disturbances do not have an immediately apparent cause. A multi-disciplinary approach involving the dedicated skills of an astute ophthalmologist, radiologist, and neurologist is paramount in averting a visual catastrophe caused by a condition highly amenable to widely available treatment.

## References

[1] Weisberg LA (1975). Benign intracranial hypertension. Medicine (Baltimore).

[2] Dandy WE (1937). Intracranial pressure without brain tumor: diagnosis and treatment. Ann Surg.

[3] Wall M, Corbett JJ (2014). Revised diagnostic criteria for the pseudotumor cerebri syndrome in adults and children. Neurology.

[4] Kesserwani H (2021). Space flight-associated neuroocular syndrome, idiopathic intracranial hypertension, and pseudotumor cerebri: phenotypic descriptions, pathogenesis, and hydrodynamics. Cureus.

[5] Wall M, Kupersmith MJ, Kieburtz KD (2014). The idiopathic intracranial hypertension treatment trial: clinical profile at baseline. JAMA Neurol.

[6] Giuseffi V, Wall M, Siegel PZ, Rojas PB (1991). Symptoms and disease associations in idiopathic intracranial hypertension (pseudotumor cerebri): a case-control study. Neurology.

[7] Digre KB, Bruce BB, McDermott MP, Galetta KM, Balcer LJ, Wall M (2015). Quality of life in idiopathic intracranial hypertension at diagnosis: IIH Treatment Trial results. Neurology.

[8] Konrad J, Vogt R, Helbig H, Oberacher-Velten I (2015). Intracranial hypertension and jugular vein thrombosis. Ophthalmologe.

[9] Thandra A, Jun B, Chuquilin M (2015). Papilloedema and increased intracranial pressure as a result of unilateral jugular vein thrombosis. Neuroophthalmology.

[10] Rohr A, Dorner L, Stingele R, Buhl R, Alfke K, Jansen O (2007). Reversibility of venous sinus obstruction in idiopathic intracranial hypertension. AJNR Am J Neuroradiol.

[11] Lee SW, Gates P, Morris P, Whan A, Riddington L (2009). Idiopathic intracranial hypertension; immediate resolution of venous sinus "obstruction" after reducing cerebrospinal fluid pressure to&lt;10cmH(2)O. J Clin Neurosci.

[12] Gbaguidi X, Janvresse A, Benichou J, Cailleux N, Levesque H, Marie I (2011). Internal jugular vein thrombosis: outcome and risk factors. QJM.

[13] Scerrati A, Menegatti E, Zamboni M, Malagoni AM, Tessari M, Galeotti R, Zamboni P (2021). Internal jugular vein thrombosis: etiology, symptomatology, diagnosis and current treatment. Diagnostics (Basel).

[14] Leach JL, Fortuna RB, Jones BV, Gaskill-Shipley MF (2006). Imaging of cerebral venous thrombosis: current techniques, spectrum of findings, and diagnostic pitfalls. Radiographics.

[15] Patel SI, Obeid H, Matti L, Ramakrishna H, Shamoun FE (2015). Cerebral venous thrombosis: current and newer anticoagulant treatment options. Neurologist.

[16] Lavorini F, Di Bello V, De Rimini ML (2013). Diagnosis and treatment of pulmonary embolism: a multidisciplinary approach. Multidiscip Respir Med.

[17] Lee Y, Siddiqui WJ (2021). Internal jugular vein thrombosis. https://www.ncbi.nlm.nih.gov/books/NBK541111/.

[18] Kearon C, Akl EA, Ornelas J (2016). Antithrombotic therapy for VTE disease: CHEST guideline and expert panel report. Chest.

